# Supplementary material for: Multimaterial 3D Printing for Arbitrary Distribution with Nanoscale Resolution
Source: Nanomaterials (Basel). 2019 Aug 2;9(8):1108. doi: 10.3390/nano9081108 (PMC6723873; doi:10.3390/nano9081108)
Supplement: Supplementary file 1 [file nanomaterials-09-01108-s001.zip › nanomaterials-553744-SI.pdf]

Article

Multimaterial 3D Printing for Arbitrary Distribution with Nanoscale Resolution

Fengqiang Zhang [1], Changhai Li [2,*], Zhenlong Wang [1,2,*], Jia Zhang [1,2] and Yukui Wang [1]

[1] School of Mechatronics Engineering, Harbin Institute of Technology, Harbin 150001, China
[2] Key Laboratory of Micro-Systems and Micro-Structures Manufacturing, Ministry of Education, Harbin Institute of Technology, Harbin 150080, China
* Correspondence: lich@hit.edu.cn (C.L.); wangzl@hit.edu.cn (Z.W.); Tel.: +86-451-8641-3485 (Z.W.)

Received: 3 July 2019; Accepted: 30 July 2019; Published: date

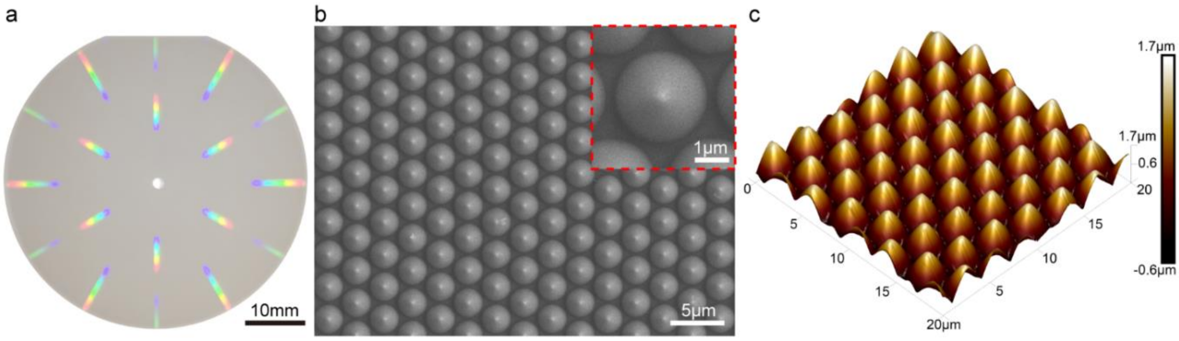

Figure S1. Patterned sapphire substrate (PSS). (**a**) The optical images of PSS illuminated by astigmatism lamp; (**b**) SEM image of PSS; (**c**), AFM image of PSS.

Table S1. Parameters of magnetron sputtering.

No.	materia l	deposition rate of W(Å/s)	sputtering power of W(W)	deposition rate of Al (Å/s)	sputtering power of Al(W)	Deposition time of each layer (s)	Thickness of each layer (nm)
1	W	5	59			480	240
2	Al			5	218	240	120
3	W	5	59			240	120
4	Al			5	218	240	120
5	W	5	59			240	120
6	Al			5	218	240	120
7	W	5	59			240	120
8	Al			5	218	240	120
9	W	5	59			240	120
10	Al			5	218	240	120
11	W	5	59			240	120
12	Al			5	218	240	120
13	W	5	59			240	120
14	Al			5	218	240	120
15	W	5	59			240	120
16	Al			5	218	240	120
17	W	5	59			240	120
18	Al			5	218	240	120
19	W	5	59			240	120
20	Al			5	218	240	120
21	W	5	59			240	120
22	Al			5	218	240	120
23	W	5	59			240	120

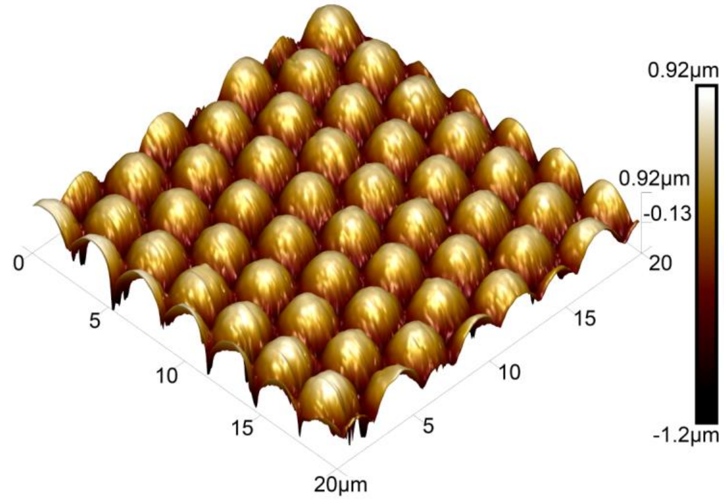

Figure S2. AFM image of lamination of multimaterials. Good uniformity of microstructures of lamination is demonstrated.

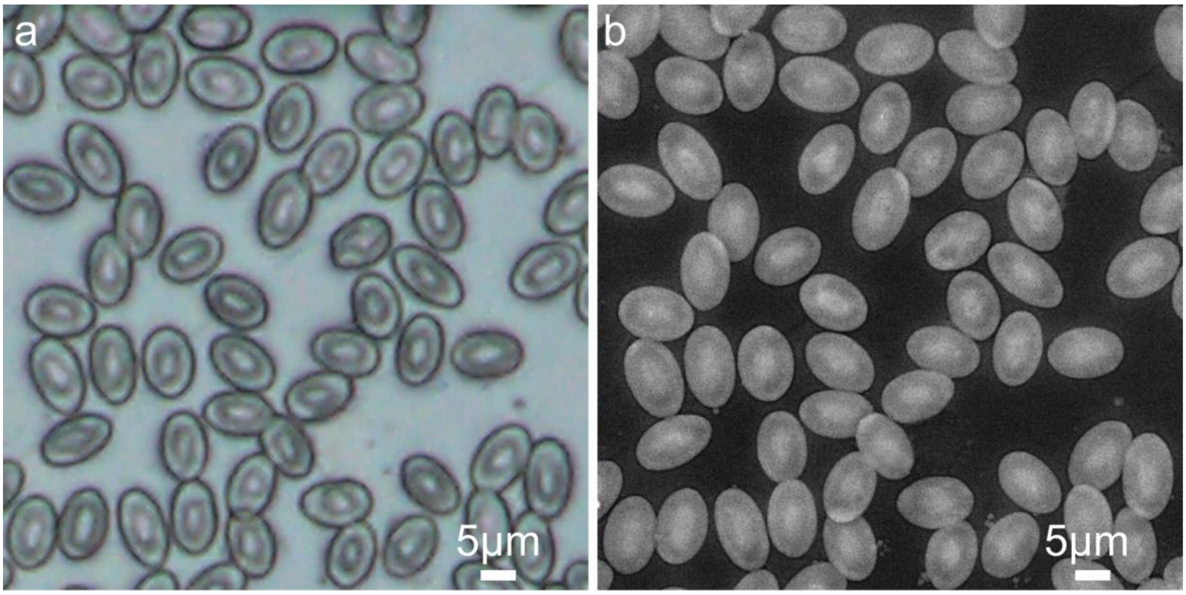

Figure S3. Comparison of the morphology of monolayer cells and the distribution of multiple materials; (**a**) Optical image of deposition surface formed by spin-coating of the erythrocytes on a quartz substrate; (**b**) SEM image of the distribution on the specified section via depositing gradient ratios of aluminum and tungsten metals on the deposition surface.
